# Inguinoscrotal region as an unusual site of extra-pancreatic collections in infected pancreatic necrosis

**DOI:** 10.1093/gastro/gou090

**Published:** 2015-02-02

**Authors:** Saurabh Kalia, Rahul Gupta, Sunil D. Shenvi, Hemanth Kumar, Rajesh Gupta, Mandeep Kang, Surinder Singh Rana, Deepak Kumar Bhasin, Rajinder Singh

**Affiliations:** ^1^Surgical Gastroenterology Division, Department of General Surgery, Postgraduate Institute of Medical Education and Research, Chandigarh, India; ^2^Department of Radiology, Postgraduate Institute of Medical Education and Research, Chandigarh, India; ^3^Department of Gastroenterology, Postgraduate Institute of Medical Education and Research, Chandigarh, India

**Keywords:** acute pancreatitis, inguinoscrotal collections, step-up approach, necrosectomy

## Abstract

Severe acute pancreatitis often leads to pancreatic and peripancreatic collections but, rarely, it can lead to collections at sites remote from the pancreas. Three male patients presented with abdominal pain and inguinoscrotal swelling. They were initially misdiagnosed with obstructed inguinal hernia, epididymo-orchitis and hydrocele, respectively. Later, their diagnosis of acute pancreatitis was revealed on laparotomy in one patient and on computed tomography (CT) in the remaining two patients. All these cases had extensive peripancreatic necrosis and paracolic collections tracking along the psoas muscle, downwards towards the pelvis. These collections were initially managed by percutaneous drainage and saline irrigation as a part of the ‘step-up’ approach. Two of these patients required open necrosectomy, while all required incision and drainage of inguinoscrotal collections. All the patients were discharged in satisfactory condition. Inguinoscrotal swelling is unusual as a first presentation of acute pancreatitis. A high index of suspicion, with careful study of patient's history and examination along with CT, may provide an accurate diagnosis. Local drainage may be required to control sepsis and also provide an egress route for intra-abdominal collections.

## Introduction

Reports of pancreatic collections, presenting at sites remote from the pancreas, are available in the literature [1, 2]. The peripancreatic collection has been reported in the form of pseudocyst in the mediastinum or pleural cavity and as swelling in the inguinal scrotal area [1–3]. The diagnosis of acute pancreatitis in such situations can be missed in the absence of classical history and physical findings. The fluid from the peripancreatic region can travel to these remote sites, traversing the fascial planes. [4] We present here three cases of inguinoscrotal swellings in patients with acute severe pancreatitis but with different presentations.

## Case presentation

### Case 1

**Figure 1.**
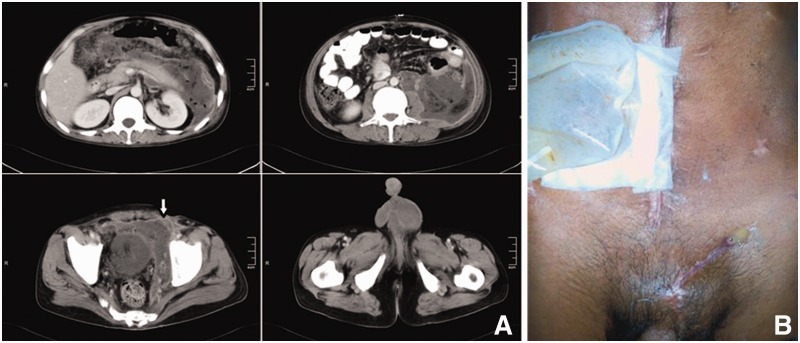
CECT scan of Case 1, showing peripancreatic tracking along combined interfascial plane into pelvis and then into left scrotum via inguinal ring (white arrow, A). Inguinal sinus with purulent discharge at site of previous inguinal exploration (post pancreatic necrosectomy and loop ileostomy status, B)

A 35-year-old male presented to a peripheral hospital with acute-onset epigastric pain radiating to the back, associated with bilious vomiting and left inguinal swelling for the previous 7 days. The patient was misdiagnosed as having an obstructed inguinal hernia and inguinal exploration was carried out. The contents of the left inguinal canal were inflamed, with haemorrhagic fluid in the inguinal canal seen to be arising from the deep inguinal ring. The patient was then converted to midline laparotomy. At exploratory laparotomy, there was fat saponification of the greater omentum and peritoneum, suggestive of acute pancreatitis. The abdomen was closed and the patient was referred to our institute. The patient's clinical history revealed that he was chronically alcoholic and had a ‘binge’ of alcohol 15 days previously, after which he had developed abdominal pain. The swelling in the inguinal region had developed later when the pain worsened. The patient was evaluated with a contrast-enhanced computed tomography (CECT) of the abdomen and pelvis. There was pancreatic necrosis with fluid collection in the peripancreatic region, left pararenal space, extending to the pelvis along the left psoas muscle. There was inflammatory fat stranding in the region of the left spermatic cord and left scrotum with oedema and bilateral pleural effusion (Figure 1A). The patient had a left inguinal wound, draining about 100 mL of dark, muddy fluid daily, and was managed with local dressings. Ultrasonography (USG)-guided pigtail catheter drainage of the lesser sac and pararenal space collections were done. The patient was managed with catheter irrigation with sterile saline, which would drain through the inguinal incision. He developed continued systemic sepsis and suspected colonic fistula through the inguinal wound. Laparotomy and pancreatic necrosectomy were done, as was a loop ileostomy and feeding jejunostomy. Closed drainage of the left retroperitoneal space and lesser sac was done. Later in the post-operative course, he developed a painful swelling in the left scrotum. On examination, the swelling was tender to palpation, fluctuant while the left testis could be felt separate from the swelling. The scrotal abscess was incised and drained brownish, necrotic material resembling peripancreatic necrosis. The patient was discharged in a satisfactory condition. Later the patient underwent sub-total colectomy for colonic fistula with inguinal sinus tract excision (Figure 1B).

### Case 2

**Figure 2.**
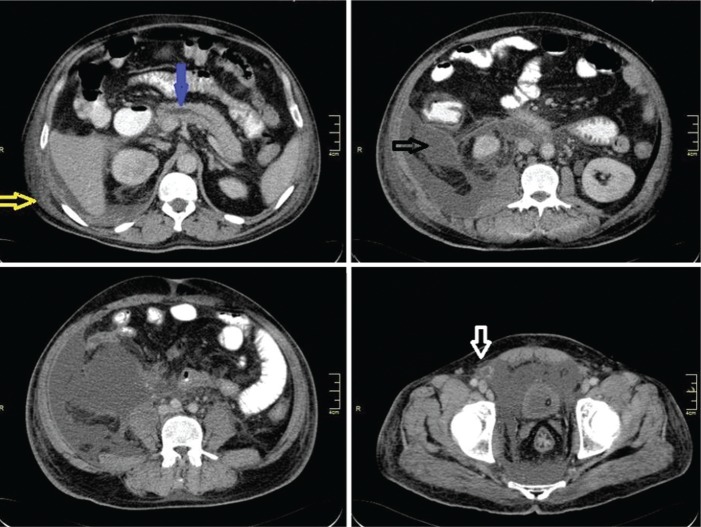
CECT scan of Case 2, showing fluid collections in the perihepatic region (yellow arrow), right anterior and posterior pararenal space and lateroconal fascia (black arrow), tracking into combined interfascial plane to pre-vesical and retrorectal space and right inguinal canal (white arrow). Dilated pancreatic duct is also seen (blue arrow).

A 48-year-old male presented with acute-onset upper abdominal pain radiating to the back for the previous 5 days. He also had abdominal distension, nausea, vomiting and a painful swelling in the scrotum. On examination, there was tenderness in the epigastrium and right lumbar region. The scrotum was swollen and tender, with inflammatory changes of the skin suggestive of acute epididymo-orchitis. A CECT of the abdomen and pelvis carried out on admission revealed a dilated main pancreatic duct with stranding and fluid collection around the uncinate process extending to the right pararenal space, right paracolic gutter and pelvis with fat stranding of the root of mesentery, retroperitoneum and scrotum (Figure 2). The patient developed spiking fever and was started on broad-spectrum antibiotics. As part of the step-up approach [5], USG-guided pigtail drainage of the right paracolic collection was done on the first day of admission, which drained nearly 1 litre of brownish (haemorrhagic) fluid, but the scrotal swelling continued to increase in size and ultrasound of the scrotum revealed a large, septated collection in the right hemiscrotum with air foci. The scrotal abscess was drained under local anaesthesia, revealing frank pus with swollen epididymis and normal testes after 12 days of pigtail drainage; however, the patient continued to have fever with worsening general condition. **Right retroperitoneal drainage of the peripancreatic- and right pararenal space necrosis and collections** was done through a flank incision 24 days after the attack of pancreatitis. Repeat CECT abdomen was done after 25 days of surgical intervention which revealed undrained collections in the pelvic retroperitoenum and, in view of persistent high-grade fever, the patient were re-explored on the next day. Midline laparotomy revealed peripancreatic necrosis tracking along the right pass to the pelvis, pre-vesical space, right lateral pelvic wall and right inguinal space. Debridement and wide drainage was done. Subsequently, the patient recovered well and was discharged in a satisfactory condition after the second surgery.

### Case 3

**Figure 3.**
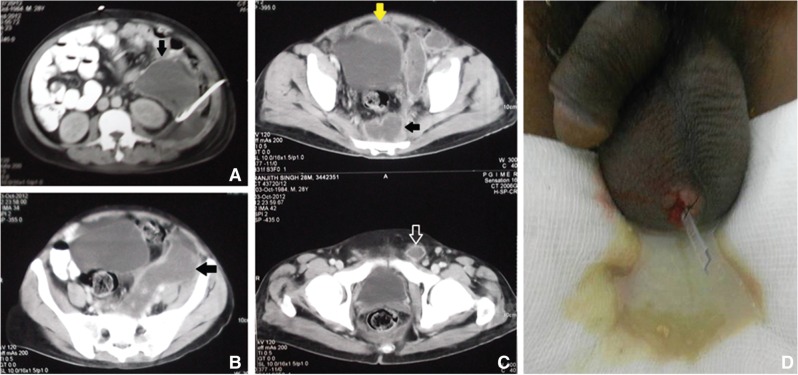
CECT scan of Case 3, showing fluid collections in the left anterior and posterior pararenal spaces (black arrow) with pigtail *in situ* (A), in the left paracolic space and extending into pelvis (black arrow, B), and in the pre-vesical space (yellow arrow), retrorectal space (black arrow) and left inguinal canal (white arrow) (C). Scrotal swelling showing turbid fluid on aspiration and after draining purulent fluid with corrugated drain insertion (D).

A 28 year old male presented with acute abdominal pain radiating to the back and breathlessness after an alcohol binge to an outside hospital 2 days after the onset. He was diagnosed with severe acute pancreatitis by CECT done at that hospital. On post pain day 8, he was referred to our Institute. He was initially managed conservatively. He developed a painful swelling in the left scrotum 10 days after onset of acute pancreatitis. USG of the scrotum showed a fluid collection in the scrotal sac with multiple septations and echogenic content. Both testes and the epididymis were normal. The fluid obtained by aspiration showed haemorrhagic fluid, had no pus cells and was sterile on culture with high amylase. Scrotal support and anti-inflammatory agents were given. After 4 weeks of conservative management, the patient continued to have a high-grade fever. A CECT showed large peripancreatic and left paracolic collections, which were drained by pigtail as part of the step-up approach (Figures 3A and 3B). The patient also received pigtail fluid culture-based antibiotics and antifungals but continued to have fever. Repeat CT of the abdomen showed a residual collection of 10 x 8 cm in the left paracolic gutter, extending into the pelvis and left scrotal sac (Figure 3c). A USG-guided pigtail was inserted into this collection. Irrigation of the collections via the pigtails was started using sterile saline and a Y-connector [5], but the patient continued to have a fever in the ninth and tenth weeks. Repeat US of the scrotum showed gross hydrocele, with thick citations and internal echoes. Incision and drainage of the scrotal collection were done under local anaesthesia at the end of the tenth week and a corrugated drain was put in place. It drained about 500 mL of purulent fluid along with necrotic debris (Figure 3D). Irrigation of percutaneous drains led to efflux from the scrotal wound and the fever subsided completely. The patient was discharged 1 week later with pigtail catheters *in situ*. On subsequent follow-up, the scrotal drain and the pigtail catheters were removed.

## Discussion

Severe acute pancreatitis manifesting as inguinoscrotal swelling is a rare presentation, causing a diagnostic dilemma [3, 4]. The fluid in peripancreatic collections can track retroperitoneally into the inguinoscrotal region, traversing the deep and superficial inguinal ring. In such a scenario it can mimic obstructed hernia, testicular torsion, acute epididymo-orchitis, hydrocele, testicular tumour, etc. A missed diagnosis may lead to unnecessary surgery and improper or delayed treatment [6, 7].

Most commonly, inflammatory fluid and necrosis extends into the anterior pararenal space and retromesenteric plane. The fluid and inflammation may spread posteriorly to involve the retrorenal plane, and laterally into the lateroconal plane, or spread inferiorly in the combined interfascial plane to reach the pelvic retroperitoneum or superiorly along the diaphragm to enter the mediastinum. These fascial planes provide a weak barrier to spreading inflammation [8]. The pelvic retroperitoneal space includes the pre-vesical space, lateral pelvic walls and retrorectal space [8]. Rarely, the fluid may then track along the deep inguinal ring, through the inguinal canal, into the scrotum. The testicle, covered by the *tunica vaginalis*, is generally spared from the inflammation.

Based on the extent of spread of peripancreatic fluid in retroperitoneal interfascial planes, Ishikawa *et al.* proposed a severity classification system for acute severe pancreatitis [9]. The grades, numbered I to V according to patterns of spread, signify more severe disease with higher morbidity and mortality with higher grades. In this classification, however, the spread of inflammatory fluid and necrosis into the inguinal region and scrotum was not separately recognized and hence was not mentioned in the classification. In this classification, the spread of inflammation and necrosis to the pelvic retroperitoneum has been categorized as Ishikawa Grade IV with higher morbidity. In the cases illustrated in this series we have shown that, if this collection from pelvic retroperitoneum spreads to inguinoscrotal region, then the treatment can actually be simpler as these inguinoscrotal collections can be managed with incision and drainage, with resolution of retroperitoneal inflammation.

The initial management of patients suffering from acute pancreatitis with an inguinoscrotal collection is similar to those without it. It involves fluid resuscitation, nutritional support, prophylactic antibiotics and intensive care in case of organ failure. In previously reported cases, this supportive care was successful in resolution of inguinoscrotal collections [3, 4]. If the patient does not respond, then pigtail drainage of the collections should be considered as a part of the ‘step-up’ approach [5]. In our experience, however, these patients required local drainage of inguinoscrotal collections for complete resolution. The liberal use of saline irrigation in our practice may also have led to an accumulation of irrigation fluid in the scrotum. Draining the scrotal collection locally actually provides an egress route for the irrigation fluid instilled through percutaneously placed drains, and probably helped in the complete resolution of retroperitoneal infected collections.

The present study supports our viewpoint that cases of inguinoscrotal swellings in severe acute pancreatitis may have variable extents of inflammation and therefore a different clinical course. Management thus has to be tailored according to the extent of spread of retroperitoneal inflammation. The value of serial computed tomography cannot be over-emphasized in this scenario. In addition, depending upon the severity of necrosis in the mesocolon, the vascularity of the affected colon may also be jeopardized, as seen in one of our cases in this series, which required sub-total colectomy.

To summarize, inguinoscrotal swelling as a first presentation of acute pancreatitis is unusual. Scrotal collections appearing in the course of acute pancreatitis may contribute to continued sepsis. A high index of suspicion with a careful study of patient's history and examination along with computed tomography of the abdomen and pelvis may provide an accurate diagnosis and extent of disease. Local drainage may help in the resolution of sepsis, both by drainage of the collection and also by providing an egress route in patients undergoing pigtail catheter irrigation of a pancreatic collection. There may still be undrained collections in other dependent areas, which require separate management for a successful outcome.

*Conflict of interest statement:* none declared.
